# Fatal cerebral arterial gas embolism after endoscopic retrograde cholangiopancreatography

**DOI:** 10.4103/0972-5229.56061

**Published:** 2009

**Authors:** Pradeep Rangappa, Britta Uhde, Roger W. Byard, Alex Wurm, Peter D. Thomas

**Affiliations:** **From:**1 Columbia Asia Referral Hospital, Yeshwantpur, Bangalore - 560 055, India; Royal Adelaide Hospital, Adelaide, SA-5000

**Keywords:** Cerebral arterial gas embolism, endoscopic retrograde cholangiopancreatography, hyperbaric oxygen (HBO_2_)

## Abstract

We report the case of a 50-year-old woman undergoing elective endoscopic retrograde cholangiopancreatography, who developed coma and hemiparesis secondary to severe cerebral artery gas embolism. Despite prompt diagnosis and early hyperbaric oxygen therapy (HBO_2_) she developed severe cerebral edema and died within 24 h.

## Introduction

The reported complication rate after endoscopic retrograde cholangiopancreatography (ERCP) is 10%, most commonly involving perforation of a viscus, hemorrhage, pancreatitis and cholangitis. Less common complications include pneumoperitoneum, retroperitoneal dissection of air, gas in the portal and hepatic veins, venous and arterial bile and air embolism.[1,2]

To the best of our knowledge, only two cases of cerebral arterial gas embolism (CAGE) during ERCP have been reported in the English literature. This case is remarkable for the fulminant clinical course and lack of response to hyperbaric oxygen therapy. Mechanisms of air embolism in the context of ERCP, diagnosis and treatment of CAGE are reviewed.

## Clinical Record

**Figure 1:**
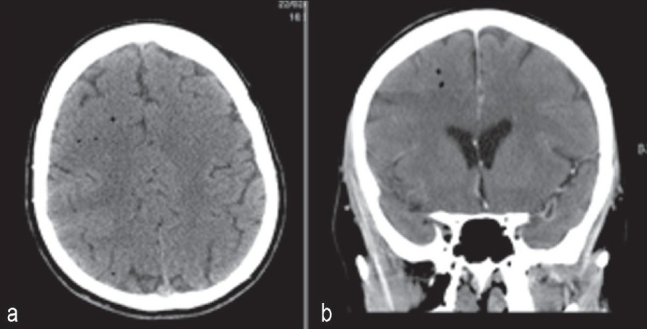
(a) CT head showing air bubbles predominantly in the right cerebral hemisphere (b) CT head showing air bubbles predominantly in the right cerebral hemisphere

A 50-year-old woman underwent elective ERCP for suspected choledocholithiasis at a suburban hospital. Three months previously she had undergone laparoscopic cholecystectomy. Subsequently an ERCP had been performed with sphincterotomy, stone extraction, and biliary stent placement. She had a past medical history of hypercholesterolemia treated with atorvastatin. ERCP was performed under sedation with 100 mcg fentanyl, 2 mg midazolam and 150 mg propofol. During the procedure, the patient was breathing spontaneously on 6 l/min of mask oxygen and was receiving Hartmann's solution through a 20G peripheral line in the dorsum of her left hand at 150 ml/hr. The procedure lasted 45 min. The stent was removed; a dilated common bile duct and an impacted stone in the cystic duct were found. Stone extraction with a balloon catheter was successful after several attempts and widening of the sphincterotomy to approximately 2.5 cm. Towards the end of the procedure, an episode of mild tachypnea was noted, but hemodynamic parameters and oxygen saturation remained normal. The patient was transferred to recovery where her vital signs remained stable but she failed to regain consciousness. Forty minutes after the procedure she was still unresponsive requiring jaw support to maintain her airway. She was noted to have a Glasgow coma score (GCS) of 5 with only a weak right-sided motor response to painful stimuli and a gaze deviation to the right. Her pupils were mid-size, equal and sluggishly reactive to light. The patient was intubated with a size 8 endotracheal tube using 100 mg ketamine and 100 mg suxamethonium. An urgent computed tomography (CT) head was organized, which revealed cerebral artery gas embolism predominantly in the right hemisphere [Figure 1].

The patient was retrieved to our tertiary hospital for hyperbaric oxygen therapy. Road transport was uneventful and sedation was maintained with a propofol infusion. On arrival to our institution, the patient was unresponsive to painful stimuli, pupils remained equal, mid-size, and reactive to light. Vital signs were stable with a mean arterial pressure (MAP) of 97 mm Hg, sinus tachycardia of 108/min, and oxygen saturation of 98% on mandatory mode of ventilation on FiO2 of 1.0, which was later weaned to FiO2 of 0.35 and spontaneous breathing mode. Cardiopulmonary examination was unremarkable; the abdomen was soft and nondistended. Blood gas analysis showed mild respiratory alkalosis and pO_2_ of 386 mmHg. Laboratory results showed a leukocytosis of 18.3 × 10^9^/l and mildly elevated alkaline phosphatase (ALP) of 108 U/l, gamma glutamyl transferase (GGT) of 96 U/l, aspartate aminotransferase (AST) of 195 U/l, alanine amino transferase (ALT) of 195 U/l and lactate dehydrogenase (LDH) of 512 U/l. The chest radiograph was normal.

**Figure 2:**
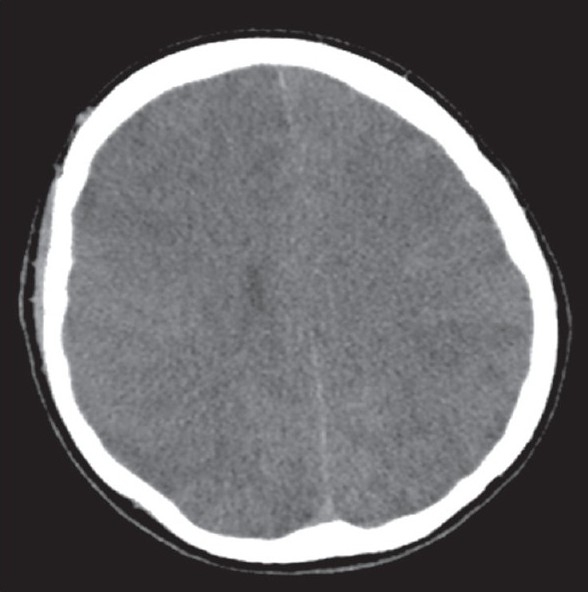
Repeat CT head showing diffuse cerebral edema but no residual intracerebral air

Bilateral myringotomies were performed, and the patient underwent hyperbaric oxygen treatment. Compression was commenced approximately 4 h after the completion of the ERCP. Pressurization was performed according to US Navy table 6 (60 ft, then 30 ft) for 4.5 h. A lidocaine infusion was commenced at 80 mg/h. After transfer to the intensive care unit, the patient remained sedated. She became febrile (38.8°C). Seven hours later, a hypertensive and bradycardic episode occurred, followed by severe hypotension and sinus tachycardia, and the pupils were now fixed and dilated. An urgent CT scan of the head was performed, which showed global cerebral edema with uncal and early tonsillar herniation. There was no residual intracranial air [Figure 2]. Shortly after, cessation of spontaneous ventilation was noted. Clinical examination and a cerebral perfusion scan confirmed brain death approximately 22 h after the ERCP.

**Figure 3:**
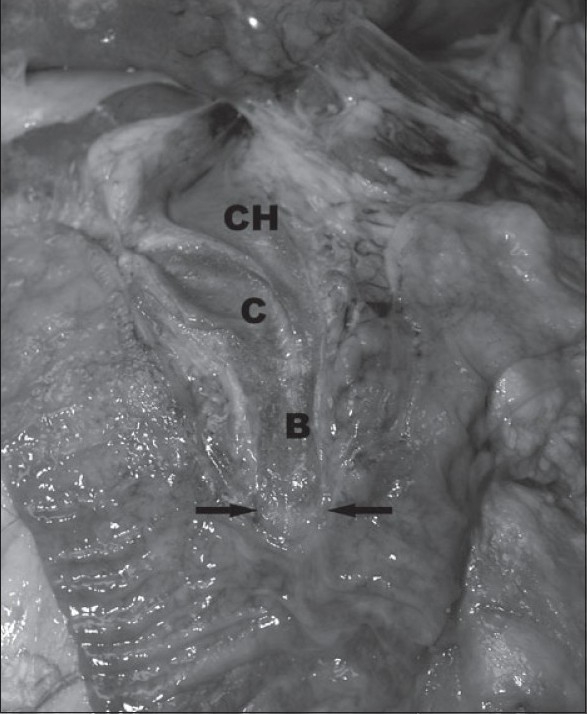
Opened duodenum and biliary tree demonstrating normal duodenal mucosa with unremarkable, although slightly, dilated bile (B), ligated cystic (C) and patent common hepatic (CH) ducts. The arrows point to the sphincterotomy site

At autopsy, there was no evidence of significant trauma at the sphincterotomy site, which was dissected *in situ* [Figure 3]. Specifically, there was no communication between the sphincterotomy site and major vessels, with unremarkable mucosa, muscularis propria and serosal surfaces of the duodenum. The common hepatic, bile and cystic ducts were patent with no evidence of stones or trauma. There were no pathological lesions such as duodenal ulceration or inflammation. Opening of the heart underwater revealed air within the right atrium with a 5-mm probe-patent foramen ovale. The brain was edematous with uncal and tonsillar herniation and changes of diffuse hypoxic ischemic damage on microscopy. Focal areas of acute myocardial ischemic injury were also noted histologically, possibly contributed by focal atherosclerotic narrowing of the left anterior coronary artery. There were no other underlying organic diseases that could have caused or contributed to death, and there was no evidence of trauma. Death was due to air embolism complicating ERCP/sphincterotomy with paradoxical embolism to the brain, resulting in significant cerebral edema and hypoxic brain injury.

## Discussion

Arterial or venous gas embolism during ERCP is very rare but is increasingly reported, as evident by the number of reports.[1] Reported risk factors for gas embolism during ERCP include disruption of mucosal integrity during sphincterotomy exposing venous radicles to air insufflation during endoscopy, and high insufflation pressure used during endoscopy.[3] Air embolism during endoscopic procedures has been related to the presence of a duodenocaval fistula,[4] a major blood vessel at the site, Crohn's disease[5] and a bleeding ulcer. Gas entry into the circulation requires two factors: an open vessel and a pressure gradient.[6] Air can be drawn into the circulation if the intravascular pressure is negative (e.g., the jugular vein in the sitting position) or forced into the vessel by positive pressure[6] as it occurs during air insufflation for endoscopy. Paradoxical embolism occurs when gas enters the arterial circulation through a right-to-left cardiac or intrapulmonary shunt or passage of bubbles occurs through the pulmonary capillary bed.[6] A patent foramen ovale is present in 33% of the normal population until the third decade and in 27% in all age groups.[7] Thirty milliliters of air entering the systemic circulation is sufficient to overwhelm the capacity of the pulmonary capillaries to filter out the air bubbles and prevent entry into the arterial circulation.[8] 0.05 ml of air in the arterial circulation is capable of causing significant neuronal damage in the brain.[9] Air bubbles more than 22 μm in diameter are normally filtered by pulmonary capillaries; however, this filter mechanism fails in the presence of a large volume of gas, barotrauma, pharmacologic agents and oxygen toxicity.[10] There are two hypotheses as to how air enters the circulation during ERCP: first, presence of severed venous radicles at the site of sphincterotomy that are exposed to high pressure gas[11] and second, the opening of functional communications between intrahepatic bile ducts and portovenous channels when exposed to high insufflation pressure during endoscopy.[12] Other cases have been linked to biliary-vascular fistulae from hepatic trauma[2] or previous invasive procedures.[1,13]

Other iatrogenic causes of CAGE reported in the literature occur in the setting of cardiopulmonary surgery, surgery of the sinuses, neck and head, laparoscopic procedures, peritoneal insufflation of air, needle biopsy of the lung, thoracocentesis, central venous cannulation, hemodialysis and arteriography.[10] The clinical features of CAGE can be quite variable although the commonest presentation is an altered sensorium and confusion.[8] Other features include dizziness, headache, seizures, coma, visual field defects, hemiparesis, paraesthesia, and cardiopulmonary arrest. Other rare signs reported are retinal gas bubbles and a pale or blue tongue (Liebermeister's sign) secondary to lingual artery embolization.[6,14]

CT scan of the brain is helpful in detecting arterial air in the acute stage, and finer cuts are found to increase the detection rate.[9,10] Air bubbles greater than 1.3 mm are generally identified in the CT brain when appropriately intersected.[6] However, absence of air on CT scan does not exclude the diagnosis. Magnetic resonance imaging (MRI), single photon emission computed tomography, or xenon-enhanced CT are only helpful in delineating regional blood flow. MRI helps in detecting areas of infarction in the acute stage, identifying areas of reversible ischemia and hemorrhagic transformation of infarcts, discriminating between acute and chronic infarcts, and identifying tissue edema in less than 1 h of an ischemic event.[10] Echocardiography may be useful in detecting gas in the ventricles and the presence of a patent foramen ovale. Other non-specific findings include elevation of creatinine kinase (CK), serum gamma-glutamyl transpeptidase, aspartate aminotransferase, and lactate dehydrogenase.[6]

The initial management of CAGE includes immediate respiratory and hemodynamic support. Trendelenburg positioning may prevent movement of arterial gas cephalad.[6] Lidocaine has been shown in animal models to improve recovery of somatosensory evoked potentials following CAGE, but its benefit in humans is questionable.[6,14] Corticosteroids have not been shown to be of any benefit and are not recommended.[14] Heparin has been shown to be of marginal benefit in animal models but cannot be recommended in patients with CAGE.[6,14] Tight control of serum glucose is advocated as this may limit the extent of neuronal damage in areas of ischemia.[6]

**Figure 4:**
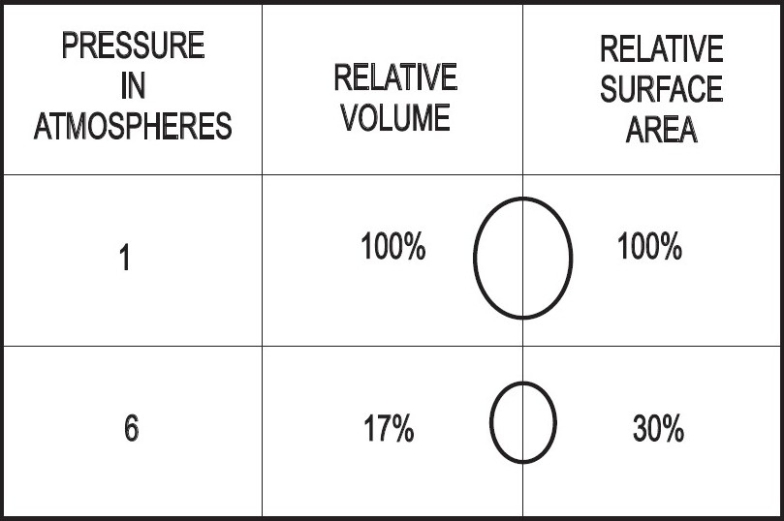
Relative volume and surface area of an air bubble with compression to six atmospheres[10]

Hyperbaric oxygen (HBO_2_) is recommended for CAGE although there are no randomized controlled trials conclusively proving its benefit.[8,14–16] One study showed a mortality rate of only 7% in 30 patients treated with hyperbaric oxygen.[17] Increased ambient pressure directly compresses the gas bubbles reducing the bubble volume and diameter in accordance with Boyle's law (volume of gas is inversely proportional to pressure at a given temperature)[6,14] [Figure 4]. HBO_2_ causes the diffusion of nitrogen from the bubble down a steeper bubble-plasma pressure gradient. All these mechanisms promote the restoration of distal blood flow. A reduction in the bubble endothelial interface decreases vascular endothelial injury and reduces the “no reflow” phenomenon, which is believed to be a cause of delayed symptom onset or failure of HBO_2_ treatment. HBO_2_ also decreases reperfusion injury by reducing leukocyte aggregation and decreasing cerebral edema.[6,14] Hundred percentage (100%) oxygen at 2.8 atm produces an alveolar pO_2_ of 2041 mm Hg. This inspired oxygen tension leads to a measured arterial pO_2_ level of up to 1800 mm Hg. These high levels of oxygen help reduce cerebral ischemia as the compressed air bubbles are being resolved and facilitate the diffusion of small air bubbles from the pulmonary capillaries to the alveoli for elimination.[6,14] There is also experimental evidence to suggest that cerebral edema is decreased by hyperbaric oxygen due to vasoconstriction caused by hyperoxia.[18] Reduction in increased intracranial pressure in severe cerebral ischemia secondary to air embolism may be an added benefit of hyperbaric oxygen therapy.[19]

In a study by Murphy *et al*., the time delay between the occurrence of air embolism and the initiation of HBO_2_ in 14 patients ranged from 10 min to 25 h, with a mean of 8.25 h.[8] The number of patients was too small to allow statistical correlation between time delay and response to therapy. However, the group judged to have a complete response averaged 6.5 h from the time of the incident to the time of therapy, and the partial responders averaged a delay of 9.5 h. Time delay was found to be 11 and 12 h in two of the three non-responders. Of the 8 patients whose time delay was 5 h or less, 5 (63%) had a complete response. Of the 6 patients whose delay in treatment was greater than 5 h, only two (33%) had a complete response. It is generally recommended that HBO_2_ should be started as quickly as possible following CAGE, and better outcomes are documented for the therapy started within 5 h.[8,12]

In our patient, the possible mechanism of CAGE was entry of gas insufflated during endoscopy into severed venous radicles at the site of sphincterotomy and subsequent entry into the arterial circulation through a patent foramen ovale.[11,12] As mentioned in other case reports, even in the absence of a patent foramen ovale, air in the venous circulation can enter the systemic circulation if the volume of air is large (>30 ml).[8] Although prompt diagnosis and appropriate treatment with HBO_2_ was started in our patient 4 h after the procedure, the outcome was poor with progression to brain death within 24 h. Prevention of cerebral air embolism by meticulous technique in the performance of an invasive procedure and terminating the procedure when there is suspicion of air in the venous system is essential.[9] It is suggested that a procedure should be terminated as soon as air is detected in the portal vein by fluoroscopy during ERCP to prevent further migration of air causing catastrophic events.[12] Early recognition of symptoms, prompt diagnosis with CT scan of the brain and transthoracic echocardiography and treatment with immediate HBO_2_ remain the key points in management of CAGE.
